# Psychometric Evaluation of the Drive for Muscularity Scale and the Muscle Dysmorphic Disorder Inventory among Brazilian Cisgender Gay and Bisexual Adult Men

**DOI:** 10.3390/ijerph20020989

**Published:** 2023-01-05

**Authors:** Cleonaldo Gonçalves Santos, Maurício Almeida, Mauro Lúcio de Oliveira Júnior, Tiffany A. Brown, Pedro Henrique Berbert de Carvalho

**Affiliations:** 1 Body Image and Eating Disorders Research Group (NICTA), Federal University of Juiz de Fora, Governador Valadares 35010-180, MG, Brazil; 2 Department of Psychological Sciences, Auburn University, Auburn, AL 36849-9027, USA; 3 Eating Disorders Program, Institute of Psychiatry (AMBULIM), University of São Paulo, São Paulo 05403-010, SP, Brazil

**Keywords:** muscle dysmorphia, drive for muscularity, measurement, psychometrics, sexual minorities, Brazil

## Abstract

Despite high levels of muscularity concerns among sexual-minority men, most of the existing literature on the drive for muscularity and muscle dysmorphia focuses on heterosexual men and has mainly been conducted in Western and English-speaking regions. The present study aimed to evaluate the psychometric properties of the Drive for Muscularity Scale (DMS) and the Muscle Dysmorphic Disorder Inventory (MDDI) in Brazilian cisgender gay and bisexual adult men who were 18–50 years old. We evaluated the factor structure of both measures using a two-step, split-sample exploratory (EFA; *n* = 704) and confirmatory (CFA; *n* = 705) factor-analytic approach, which supported the original three-factor structure of the MDDI and resulted in a reduced two-factor solution with 13 items for the DMS. Convergent validity was supported through associations of the DMS and the MDDI with eating disorder symptoms, body-ideal internalization, self-objectification beliefs and behaviors, and body appreciation measures. Additionally, we found good internal consistency, and test–retest reliability of both measures. Results support the validity and reliability of the DMS and the MDDI in Brazilian cisgender gay and bisexual adult men and will support future studies exploring these constructs in Brazilian sexual-minority men.

## 1. Introduction

Muscle dysmorphia (MD) is characterized by a pathological preoccupation with one’s degree of muscularity that involves distress and fear over the idea that one’s body is too small or not sufficiently muscular [1]. Individuals with MD engage in rigid and obsessive behaviors that can have serious health consequences and impair psychosocial functioning [1]. Specifically, individuals with MD display attitudes and behaviors similar to people with a high drive for muscularity, including excessive exercise, muscularity-oriented disordered eating, and the use of performance-enhancing drugs (e.g., androgenic anabolic steroids) [2,3,4]. Indeed, a systematic review with meta-analysis pointed to the drive for muscularity as a risk factor for MD [5]. Furthermore, research using non-clinical samples has demonstrated associations between drive for muscularity and MD symptoms such as higher eating disorder (ED) symptoms [6,7,8,9], body-ideal internalization [10], depressive symptoms [11], self-objectification, and exercise dependence [12]. Moreover, higher muscularity concerns have been associated with lower self-esteem [13] and body appreciation [14,15].

Several instruments have been developed to study and assess MD symptoms in the absence of a clinical interview (i.e., using the Diagnostic and Statistical Manual of Mental Disorders, Fifth Edition (DSM-5) diagnostic criteria), including the Adonis Complex Questionnaire [16], the Muscle Dysmorphia Symptom Questionnaire [17], the Muscle Appearance Satisfaction Scale [18], the Muscle Dysmorphia Inventory [19], the Muscle Dysmorphic Disorder Inventory (MDDI) [20], the Muscle Dysmorphia Scale [21], and the Muscle Dysmorphia Symptom Questionnaire [22]. Systematic reviews found that the MDDI is among the most widely used screening tools specific to MD [5,23]. The MDDI assesses various nosological aspects of MD [20]. It includes *Appearance Intolerance* (AI; i.e., negative beliefs about one’s body and the resulting appearance anxiety or body exposure avoidance), *Drive for Size* (DFS; i.e., thoughts of being smaller, less muscular, and weaker than desired, or wishes to increase size and strength), and *Functional Impairment* (FI; i.e., impairment associated with the symptoms of MD). Moreover, the MDDI is the only screening instrument that assesses FI, a core component of the DSM-5 diagnostic criteria of MD [1].

In the MDDI original validation study, Hildebrandt et al. [20] found a three-factor solution (i.e., AI, DFS, and FI) with 13-items in a sample of U.S. male weightlifters, aged 18–72. The MDDI and its factors had good one-week test–retest reliability and internal consistency (Cronbach’s α ranged from 0.77 to 0.85). The MDDI total score significantly correlated with measures of drive for bulk, bulimic symptomatology, body dissatisfaction, obsessive compulsive symptomatology, social physique anxiety, and a measure designed to obtain general information about weightlifting, dietary practices, and behaviors related to appearance control [20]. Among prior validation studies conducted that included a factor-analytic approach, all but one [24] replicated the original three-factor structure of the MDDI proposed by Hildebrandt et al. [20].

Clinical and epidemiological research support that MD symptoms have been associated with drive for muscularity [4]. The Drive for Muscularity Scale (DMS) is one of the most commonly used tools to assess attitudes and behaviors regarding preoccupation with increasing muscularity [25]. McCreary et al. [26] evaluated the factor structure of the DMS in a sample of Canadian male high school and college students, aged 13–78, and found a two-factor solution (Muscularity-Oriented Body Image (MBI) and Muscularity-Oriented Behaviors (MB)) comprised 15 items. It is worth mentioning that one item (#10: “I think about taking anabolic steroids”) was omitted from the factor calculations [26]. However, in some studies (e.g., McPherson et al. [27]), item #10 was included in the DMS MB subscale. Since its initial validation, the DMS has been used among men and women of many countries, cultures, languages, and ages [28].

It is worth mentioning that the MDDI and DMS validation studies mostly include men regardless of their sexual orientation. For the MDDI, only three previous studies specifically examined the psychometrics in sexual-minority men or gender minorities [7,8,9]. Compte et al. [8] assessed the psychometric properties of the MDDI in a sample of cisgender gay men living in the U.S. or its territories, aged 18–50, replicating the original three-factor solution. The three-factor solution was also confirmed by Nagata et al. [9] in a study with transgender men living in the U.S. or its territories, aged 18–67, and by Compte et al. [7] in a study with gender-expansive individuals (i.e., people whose gender identity differs from that assumed for their sex assigned at birth and is not exclusively binary man or woman) living in the U.S. or its territories, aged 18–74. 

Likewise, for the DMS, only three studies focused specifically on sexual-minority men [10,29,30]. Deblaere and Brewster [29] evaluated the psychometric properties of the DMS in a mixed sample of U.S. sexual-minority men (79% gay men) aged 18–62, replicating the original two-factor solution (omitting item #10 [26]). The two-factor solution was also confirmed by Klimek et al. [30] in a study with U.S. cisgender sexual minority young adult men, and by Nerini et al. [10] in a study with Italian gay adult men. Item #10 was also omitted in the study of Nerini et al. [10]. Furthermore, according to Klimek et al. [30] this item showed poorer statistical and descriptive fit in sexual-minority men, compared with the 14-item factor structure (excluding item #10).

Notably, there is limited research in this area, and the present investigation could help to support more research into muscularity concerns and MD symptoms among sexual-minority men—an at-risk population. Indeed, there has been a dearth of research on MD in non-heterosexual men, despite increasing evidence of elevated muscularity concerns among sexual-minority men, including drive for muscularity [8,31]. For example, a review that included five studies composed of more than 100,000 participants found that gay men were more likely than heterosexual men to report dissatisfaction with their physical appearance and muscle size/tone; and experience objectification, surveillance, appearance-based social comparison, and pressure from the media to be attractive [32]. These results demonstrate that sexual orientation is an important factor to be considered in studies on body image and MD in men.

The studies by Convertino et al. [33] and Oshana et al. [34] support that this increase, experienced by sexual-minority men may be in part due to minority stressors, such as fear of rejection, sexual orientation concealment, internalized homophobia, stigma, prejudice, and discrimination. Interestingly, recent research found that minority stressors can impair one’s self-perception and may be risk factors for body dysmorphic disorder, including MD [34]. Again, these results indicate that sexual-minority men have idiosyncrasies in relation to their body image and physical appearance concerns which should be considered in validation studies.

In addition to the small number of studies on muscularity concerns in non-heterosexual men, there is a paucity of studies on drive for muscularity and MD symptoms in non-Western or non-English-speaking countries. In Latin America, although anti-discrimination laws exist, cisgender gay and bisexual men have suffered from high rates of discrimination and violence [35,36]. Furthermore, researchers have suggested that Brazilian culture promotes a “cult of the body” attributing importance to physical appearance and sculpted bodies for sexual-minority men and men regardless of sexual orientation alike [37]. For example, in a study conducted with Brazilian gay men (*n* = 646), 69.7% of the participants reported body image dissatisfaction [38]. Research also supports that body-dissatisfied gay men are more likely to engage in health risk behaviors, such as condomless anal sex [39], anabolic-androgenic steroid use [9], and disordered eating [40]. In a prior study, it was highlighted that Brazilian gay and bisexual men have a higher prevalence of mental health concerns when compared to Brazilian heterosexual men, causing greater demand for mental health services in this population [41]. 

Therefore, the objectives of the current study were: (1) to examine the factor structure of the MDDI and the DMS, using a two-step, split-sample exploratory (EFA) and confirmatory (CFA) factor analytic approach, in a sample of Brazilian cisgender gay and bisexual adult men; (2) to evaluate the convergent validity of the MDDI and the DMS with ED symptoms, self-objectification beliefs and behaviors, body-ideal internalization, and body appreciation measures; and (3) to estimate the internal consistency and two-week test–retest reliability of the MDDI and the DMS in a sample of Brazilian cisgender gay and bisexual men. It was hypothesized that the DMS [25,26] and MDDI [20] would replicate the original two- and three-factor structure, respectively (Hypothesis 1). Further, it was also expected that the DMS and MDDI would be positively correlated with measures of ED symptoms, self-objectification beliefs and behaviors, and body-ideal internalization (Hypothesis 2). Conversely, it was anticipated that the DMS and MDDI would be negatively correlated with body appreciation (Hypothesis 2). Finally, adequate internal consistency and good test–retest reliability were expected for both measures (Hypothesis 3). 

## 2. Materials and Methods

### 2.1. Participants and Procedures

As part of a larger study, the present study aimed to evaluate the psychometric properties of body image and MD measures among Brazilian cisgender gay and bisexual adult men. A total of 1418 Brazilian cisgender gay and bisexual adult men participated in the current study. For conducting EFA and CFA, a 20:1 participant per item ratio was used [42]. Furthermore, after the two-week period, a random subset of participants (*n* = 188) were selected to respond to the DMS and the MDDI to examine test–retest reliability.

A relevant institutional review board was responsible for the ethical approval of the present study (Universidade Federal de Juiz de Fora, Brazil, approval number 4.690.224), and all procedures were in accordance with the principles specified in the Declaration of Helsinki. Potential participants were invited through websites, social networking, and online communities. Including the title of the study, the advertisement invited Brazilian cisgender gay and bisexual adult men to participate in a study on body appearance concerns. Specific inclusion criteria were: (a) being a Brazilian citizen, (b) self-identified as a cisgender gay or bisexual man, (c) aged 18–50, and (d) having the ability to read and respond to a questionnaire written in Brazilian Portuguese. Exclusion criteria (i.e., having any medical condition that may directly or indirectly influence one’s physical appearance, including rheumatic or autoimmune diseases, cancer, or severe burns) was used to approximate sampling criteria used in prior Brazilian studies [43]. 

Data were collected on a cloud-based, web-responsive, secure platform accessible from any smartphone, tablet, or computer (i.e., Google Forms), from August to December 2021. A digital informed consent was provided by the participants and after they responded to the research protocol (measures described below). In order to control order effects, the scales were counterbalanced. Participants were volunteers and did not receive any benefit for participating in the study. To ensure that participants responded only once to the survey protocol, IP addresses (internet protocol) were controlled, not allowing access more than once.

### 2.2. Measures

#### 2.2.1. Demographic Data

Sociodemographic information was based on self-reporting and included: (a) age, (b) race/ethnicity, (c) gender identity, (d) sexual orientation, (e) body mass, and (f) height. The body mass index (BMI) was calculated through body mass (kilograms) divided by height (meters) squared [44]. The following categories were used to classify race/ethnicity: White (*branco*), Brown (*pardo* or “Mixed”), Black (*preto*), Yellow (*amarelo*), Indigenous (*indígena*), and “Other” (*outro*) [45].

#### 2.2.2. Drive for Muscularity Scale (DMS)

The DMS is a 15-item self-report measure that aims to assess muscularity-oriented attitudes and behaviors [25]. The DMS factor structure is composed of two subscales: *Muscularity-Oriented Behaviors* (MB; 7 items) and *Muscularity-Oriented Body Image* (MBI; 8 items) [25,26]. The Brazilian version of the DMS [46] showed adequate factor validity, good internal consistency, and satisfactory evidence of convergent and discriminant validity, and was used in the present study. It is noteworthy that the factorial structure of the DMS proposed by Campana et al. [46] is composed of only 12 items and two subscales, MBI and MB. However, in the current study, the 15 Brazilian translated items were used [46]. Each item is scored on a six-point Likert-type scale (1 = always to 6 = never). Subscale scores are derived from the sums of all items that compose them. Scores for each item were reversed to calculate the subscale scores; higher scores reflect greater muscularity-oriented attitudes and behaviors.

#### 2.2.3. Muscle Dysmorphic Disorder Inventory (MDDI)

The MDDI is a 13-item self-report instrument designed to assess core MD attitudes and behaviors [20]. The MDDI factor structure is composed of three subscales, in which the *Drive for Size* (DFS) subscale is represented by five items, and the *Functional Impairment* (FI) and *Appearance Intolerance* (AI) subscales are represented by 4 items each. The Brazilian version of the MDDI [47] upheld the original factor structure and showed good convergent validity, internal consistency, and test–retest reliability, and was applied in the current study. Each MDDI item is scored using a five-point Likert-type scale (1 = never to 5 = always). The score for each subscale is obtained by the sum of all the items that compose them. Higher scores indicate greater core MD attitudes and behaviors.

#### 2.2.4. Eating Disorder Examination-Questionnaire (EDE-Q)

The EDE-Q is a 28-item self-reported measure developed to assess ED symptoms [48]. A set of 22 items are rated on a seven-point Likert-type scale (0 = no days/none of the times/not at all to 6 = every day/every time/markedly), and six items assess the frequencies of behaviors (i.e., items 13 to 18) occurring during the past 28 days [48]. The Brazilian Portuguese version of the EDE-Q was used in the present study [49,50]. The global score may be obtained by the average of the items. Higher scores are indicative of greater ED symptoms. In the present study, the EDE-Q demonstrated good internal consistency (McDonald’s omega (*ω*) = 0.92 (95% confidence interval [CI] = 0.91, 0.93)).

#### 2.2.5. Sociocultural Attitudes towards Appearance Questionnaire—4 Revised (SATAQ-4R)

The SATAQ-4R, its male version, is a 28-item self-report measure developed to assess appearance-ideal internalization and appearance pressures [51]. The Brazilian SATAQ-4R appearance-ideal internalization subscales (i.e., *Internalization: Thin/Low Body Fat* (BF), *Muscular* (MUS), and *General Attractiveness* (GA)) were used in the present study [52]. The subscale items are scored on a five-point Likert-type scale (1 = definitely disagree to 5 = definitely agree). The SATAQ-4R appearance-ideal internalization subscales score is obtained by the average of the items that compose it. Higher scores are indicative of greater appearance-ideal internalization. For the GA subscale, items #9 and #14 were scored in reverse. Regarding internal consistency, the SATAQ-4R MUS subscale proved to be adequate (*ω* = 0.93 (95% CI = 0.92, 0.94)). The inter-item correlation was performed to calculate the reliability of the SATAQ-4R BF (*r* = 0.84) and the SATAQ-4R GA (*r* = 0.72) subscales, given both have only two items each [53].

#### 2.2.6. Self-Objectification Beliefs and Behaviors Scale (SOBBS)

The SOBBS is a 14-item self-report measure developed to assess self-objectification beliefs and behaviors [54]. The Brazilian SOBBS subscales: *Observer’s Perspective* (OP) and *Valuing the body above other attributes and qualities and the body capable of representing itself* (VB), were used in the present study [52,54]. The subscales items are scored on a five-point Likert-type scale (1 = strongly disagree to 5 = strongly agree), and each SOBBS subscales score is calculated by averaging the items. Higher scores indicate greater self-objectification beliefs and behaviors [52]. In the current study, the SOBBS subscales showed good internal consistency (SOBBS-OP subscale, *ω* = 0.91 (95% CI = 0.90, 0.92); SOBBS-VB subscale, *ω* = 0.85 (95% CI = 0.83, 0.86)).

#### 2.2.7. Body Appreciation Scale-2 (BAS-2)

The BAS-2 is a 10-item self-report instrument designed to assess body appreciation [55]. The items are scored on a five-point Likert-type scale (1 = never to 5 = always). The total score is derived from the average of all items. Higher scores are indicative of greater body appreciation. The Brazilian version of the BAS-2 showed good factor validity (i.e., EFA and CFA), convergent validity, good internal consistency, and two-week test–retest reliability for a one-dimensional structure [15,56]. In the present sample, the BAS-2 showed adequate internal consistency (*ω* = 0.94 (95% CI = 0.93, 0.95)).

### 2.3. Data Analyses

#### 2.3.1. Descriptive Statistics

Following recommendations by Parent [57], 9 participants were excluded because they were missing substantial (i.e., >80%) item-level information. Next, 0.41% (DMS, *n* = 86) and 0.38% (MDDI, *n* = 70) of item-level data were missing completely at random, as determined by Little’s (1988) Missing Completely at Random test (DMS = χ^2^ [342] = 311.62, *p* = 0.87; MDDI = χ^2^ [172] = 204.82, *p* = 0.06). The missing data were replaced using expectation maximization method [57]. Following good practices for validating measurements, an EFA-to-CFA analytic approach was used [58]. Thus, the final dataset (*n* = 1409) was divided into two random samples (EFA, *n* = 704; and CFA, *n* = 705).

The categorical variables were described by relative and absolute frequencies, and the numerical data were described by means (*M*) and standard deviations (*SD*). Univariate (i.e., asymmetry < 3 and kurtosis < 7) and multivariate normality (i.e., Mardia’s coefficient < 5) were assessed [42]. To compare the sociodemographic data between the EFA and CFA samples and between CFA and retest samples, the chi-squared test and Student *t* test were used [42].

#### 2.3.2. Factor Structure

EFAs with principal-axis factoring and oblique promax rotation were conducted to explore the factor structure of both the DMS and the MDDI [42]. The Kaiser–Meyer–Olkin (KMO > 0.80) and the Bartllet’s sphericity test (*p* < 0.05) were performed to identify the suitability of the data for factor analysis [42]. To decide on the number of factors to retain in each EFAs, we used multiple criteria, including the Kaiser–Guttman criterion (eigenvalue > 1), examination of the scree plot, and parallel analysis [59]. The factor loadings (*λ*) matrix was analyzed to identify the correspondence of the items with their respective factors, in which values ≥ 0.40 were considered adequate. Only items with *λ* ≥ 0.32 were considered cross-loading [58].

Using data from the second half-sample, CFAs with the weighted least square mean and variance adjusted (WLSMV) were performed to confirm each factor structure previously identified for the DMS and the MDDI. The existence of multivariate outliers was explored by the Mahalanobis squared distance (D^2^). The model’s adequacy [58] was evaluated by the Chi-squared test weighted by degrees of freedom (*χ*^2^/*df* < 3), root mean-square error of approximation (RMSEA < 0.08; 90% CI; *p* > 0.05), comparative fit index (CFI; values close to 0.95), Tucker–Lewis index (TLI; values close to 0.95), and standardized root mean-square residual (SRMR < 0.08). The model adjustment was performed using the Lagrange multipliers when the score was greater than 11 [59].

#### 2.3.3. Convergent Validity

The convergent validity of both measures was examined via Spearman’s rank order correlation coefficient (*rho*) among the DMS (i.e., the MB and MBI subscales) and the MDDI (i.e., the DFS, AI, and FI subscales) scores with ED symptoms (EDE-Q), body-ideal internalization (i.e., the SATAQ-4R BF, SATAQ-4R MUS, and SATAQ-4R GA subscales), self-objectification beliefs and behaviors (i.e., the SOBBS OP and SOBBS VB subscales), and body appreciation (BAS-2) measures. Following Cohen [60] cut-offs, correlations between 0.10–0.29 were considered small, correlations between 0.30 and 0.49 were considered moderate, and correlations above 0.50 were considered large. 

#### 2.3.4. Internal Consistency and Test–Retest Reliability

To estimate the internal consistency of the measures, McDonald’s omega (*ω*) was used, whose values of 0.70 or higher were considered acceptable internal consistency [61]. Using a two-week interval, test–retest reliability was evaluated through Spearman’s *rho* and intraclass correlation coefficient—ICC [59]. Correlations of 0.10–0.29, 0.30–0.49, and above 0.50 were considered small, moderate, and large, respectively [60]. Following Koo and Li [62] cut-offs, ICC values greater than 0.90 were considered to show excellent reliability, between 0.75 and 0.90 were considered good, between 0.50 and 0.75 were considered moderate, and less than 0.50 were considered poor.

All analyses were conducted with JASP version 0.16.3.0 (JASP team, University of Amsterdam, Netherlands) [63] and used a significance level of *p* < 0.05.

## 3. Results

### 3.1. Descriptive Statistics

Demographic data regarding age, BMI, race/ethnicity, and sexual orientation for the two half-samples (i.e., EFA and CFA) can be seen in Table 1. Regarding demographic data, no statistically significant differences were found between EFA and CFA samples (*p*s > 0.05). Participants (*n* = 188; 159 gays and 29 bisexual men) who responded to the two-week retest for the DMS and MDDI (i.e., retest sample; *M*_age_ = 27.06, *SD* = 5.60) self-identified themselves as White (60.63%), Brown (26.06%), Black (11.70%), and Other ethnic origins (1.61%). The BMI of the participants from the retest ranged from 17.49 to 45.84 kg/m² (*M* = 25.97, *SD* = 5.70). No statistically significant differences were found between the CFA and retest samples (*p*s > 0.06).

### 3.2. Factor Structure

Regarding the DMS, the KMO (0.89) and the significance of Bartllet’s sphericity test (*χ*^2^ [105] = 6440.736, *p* = 0.001) showed adequate common variance to conduct the factor analysis. All retention criteria (i.e., eigenvalue, scree plot, and parallel analysis) showed that the two-factor structure was the most appropriate. Parallel analysis showed that item #12 (i.e., “I think that my weight-training schedule interferes with other aspects of my life”) did not have adequate loading in its respective factor (*λ* = 0.29). Additionally, item #9 (i.e., “I think that I would look better if I gained 10 pounds in bulk”) showed cross-loading. After excluding both items, the DMS presented an adequate two-factor structure (KMO = 0.89; Bartllet’s sphericity test (*χ*^2^ (78) = 5919.507, *p* = 0.001)). The factor loadings, eigenvalues, and the total variance explained are described in Table 2.

Regarding the MDDI, the KMO (0.79) and the significance of Bartllet’s sphericity test (*χ*^2^ [78] = 4020.732, *p* = 0.001) indicated good suitability to factor analysis. All retention criteria demonstrated that a three-factor structure was the best fit to the data, replicating the original structure [20]. The factor loadings, eigenvalues, and the total variance explained are described in Table 3.

No multivariate outliers were identified. We conducted CFAs with the second half-sample for both the DMS and the MDDI. The results from CFA confirmed the two-factor solution of the DMS, showing good fit to the data: *χ*²/*df* = 5.17; CFI = 0.98; TLI = 0.97; RMSEA = 0.077 (90% CI =.069, 0.085; *p* > 0.05); and SRMR = 0.072. Furthermore, the results from CFA confirmed the three-factor solution of the MDDI, showing acceptable fit to the data: *χ*²/*df* = 6.52; CFI = 0.94; TLI = 0.92; RMSEA = 0.089 (90% CI =.080, 0.097; *p* > 0.05); and SRMR = 0.085. Standardized factor loading estimates for both the DMS and the MDDI were all adequate (see Supplementary Materials, Figures S1 and S2). Furthermore, it was decided not to use the modification indices.

### 3.3. Convergent Validity

The convergent validity of the DMS and the MDDI were performed with the CFA sample (Table 4). The DMS MBI subscale demonstrated positive and large associations with the MDDI DFS, SATAQ-4R MUS, and SOBBS OP subscales. Moreover, the DMS MBI subscale was positively and moderately correlated with the MDDI FI, SOBBS VB, and SATAQ-4R GA subscales, and correlated with the EDE-Q with a small, positive effect. Finally, the DMS MBI subscale demonstrated a negative, moderate association with the BAS-2.

The DMS MB subscale exhibited positive and large correlations with the MDDI FI and SATAQ-4R MUS subscales. In addition, a positive and moderate association was found between the DMS MB subscale and the MDDI DFS subscale. In addition, positive and small associations were found between the DMS MB subscale and the SATAQ-4 GA, SOBBS OP, and SOBBS VB subscales. Finally, a positive and small association was found between the DMS MB subscale and the EDE-Q.

In turn, the MDDI DFS subscale showed a positive and large association with the SATAQ-4R MUS subscale. Furthermore, a positive and moderate association was found between the MDDI DFS subscale and the SOBBS OP subscale, and positive and small associations with the SOBBS VB and the SATAQ-4R GA subscales. Finally, the MDDI DFS subscale showed moderate negative associations with the SATAQ-4R MUS subscale and the BAS-2.

The MDDI AI subscale showed large positive associations with the EDE-Q and the SOBBS OP subscale. Furthermore, moderate positive associations were found between AI and the SATAQ-4R BF and the SOBBS VB subscales, and small positive associations were found with the SATAQ-4R GA and the SATAQ-4R MUS subscales. The MDDI AI subscale showed a negative and large association with the BAS-2.

The MDDI FI subscale showed positive and moderate associations with the SATAQ-4R MUS and the SOBBS OP subscales, and the EDE-Q. Positive and small associations were found between the MDDI FI subscale and the SOBBS VB and the SATAQ-4R GA subscales. Finally, the MDDI FI showed a negative and small association with the BAS-2. 

### 3.4. Internal Consistency and Test-Retest Reliability

The DMS and the MDDI demonstrated adequate internal consistency for the total and subscale scores, and good test–retest reliability, showing strong association between the test and retest scores. These results are presented in Table 5.

## 4. Discussion

Cisgender gay and bisexual men have been mostly neglected in the MD literature, despite evidence of greater levels of body dissatisfaction and drive for muscularity [8,31]. Perhaps, this is in part due to uncertainty regarding the applicability and utility of existing measures cross-culturally [8,31]. Thus, in this study we evaluated the psychometric properties of the DMS and the MDDI among Brazilian cisgender gay and bisexual adult men. Results from EFAs and CFAs confirmed the original three-factor solution for the MDDI [20] and resulted in a reduced two-factor solution with 13 items (excluding items #9 and #12) for the DMS [26], respectively. Additionally, the DMS and the MDDI showed evidence of convergent validity, as well as good internal consistency and two-week test–retest reliability.

As previously hypothesized, our results supported the original three-factor solution of the MDDI, (i.e., DFS, AI, and FI; [20]) with all 13 items. These findings have been shown to be stable for many countries. For instance, a previous validation study with physically active Brazilian college men [47] and sexual minorities (i.e., living in the U.S. or its territories) was evaluated in three different studies. Specifically, cisgender gay men [8], gender-expansive individuals [7], and transgender men [9] were analyzed. Interestingly, our results confirm previous data from MDDI validation studies, which suggests stability of the factorial structure of the MDDI, regardless of gender identity and sexual orientation.

Results also confirm the original factor structure of the DMS [26], with the exclusion of items #9 and #12 in the EFA, resulting in a reduced two-factor structure with 13 items, which was then confirmed through CFA. Previous validation studies of the DMS among sexual-minority men have confirmed the two-factor structure with 14-items [10,29,30]. However, previous validation studies with men (i.e., men regardless of sexual orientation) have failed to confirm the original factor structure of the DMS with all 14 items [46,64,65]. For example, the Canadian version of the DMS was tested in recreational weightlifters and non-weightlifters and resulted in a reduced version after excluding items #10 and #15 [65]. Keum et al. [64] found support for a reduced version of the DMS for Asian American men after excluding items #4, #5, and #10. Specifically, a previous validation study with Brazilian men [46] resulted in a reduced version, after excluding items #7 (i.e., “I feel like I have too much body fat”), #9 (i.e., “I think that I would look better if I gained 10 pounds in bulking”), and #10 (i.e., “I think about taking anabolic steroids”). Our results indicated that item #9 may be particularly problematic in the Brazilian context. However, the reasons for the different findings across cultures are not clear. Maybe, some items of the DMS function differently for Brazilian men regardless of their sexual orientation [46], and Brazilian gay and bisexual men. Future studies may benefit from the analysis of measurement invariance of the DMS between heterosexual and sexual-minority men.

Regarding item #10 (i.e., referring to the use of anabolic steroids), the original DMS study advocates that the item did not load significantly on any of the lower-order factors [26]. McCreary et al. [26] suggested that item #10 can be included or deleted from the DMS at the researchers’ discretion; however, when included, it should not be considered in the calculation of the overall DMS score. For example, in the study of McPherson et al. [27], item #10 loaded into the DMS MB subscale. All available validation studies of the DMS among sexual-minority men have omitted item #10 [10,29,30]. In particular, the Brazilian version of the DMS by Campana et al. [46] chose to omit item #10 because of their high residuals. Deblaere and Brewster [29] suggested that investigators allow the scope and intention of their research questions to guide their decision regarding the retention or exclusion of item #10, and that future studies should evaluate the validity of the 15 items of the DMS with sexual-minority men. In the present study, we chose to evaluate all 15 items of the DMS. We kept item #10 in EFA due to its satisfactory factor loading (*λ* = 0.43) in its respective MB factor. CFA confirmed that item #10 should be retained.

Confirming our second hypothesis, the MDDI and DMS demonstrated good convergent validity, as evidenced through associations with ED symptoms, self-objectification beliefs and behaviors, body-ideal internalization, and body appreciation. Eik-Nes et al. [11] found an association between drive for muscularity and ED symptoms in a sample of 2460 gay, bisexual, and heterosexual men. As in our findings, Martins et al. [66] found a positive correlation between the drive for muscularity and self-objectification in gay and heterosexual men. Nerini et al. [10] validated the DMS for Italian heterosexual and gay men, finding convergent validity of the DMS with a measure of body-ideal internalization. Moreover, Alleva et al. [14] found small, negative correlations between body appreciation and drive for muscularity among gay and heterosexual men. 

Regarding MD symptoms, the MDDI scores exhibited positive and small to strong associations with ED symptoms, self-objectification beliefs and behaviors, and body-ideal internalization. Moreover, the MDDI scores demonstrated a moderate, negative association with body appreciation. Our results are consistent with a recent meta-analysis that showed that MD and ED symptoms are positively associated (*r* = 0.36; 95% CI =.30, 0.41) [6]. Prior studies have also confirmed that MD symptoms are associated with self-objectification [39]. Furthermore, Klimek et al. [67] found a positive association between body-ideal internalization and MD symptoms in gay, bisexual, and straight men. Taken together, these results support the convergent validity of the DMS and the MDDI among Brazilian cisgender gay and bisexual men.

Both the DMS and the MDDI demonstrated good internal consistency and adequate two-week test–retest reliability, confirming our third hypothesis. The internal consistency of the MDDI scores found in the present study is consistent with finding from Compte et al. [8], which evaluated gay men living in the U.S. or its territories (*ω* ranging from 0.77 to 0.89). Although evaluating temporal stability of a new measure is an important and indispensable step [58], none of the previous validation studies of the MDDI conducted with sexual-minority men [7,8,9] have examined the temporal stability. Results from the present study support that MDDI scores demonstrated good test–retest reliability, supporting the use of the MDDI across time to assess MD symptoms in Brazilian cisgender gay and bisexual adult men. 

The internal consistency of the DMS scores was good in the present study, with similar values found in previous studies with sexual-minority men from the U.S. [29,30]. It is worth mentioning that none of previous validation studies of the DMS with sexual-minority men [10,29,30] evaluated the test–retest reliability of the measure. Thus, the current study provides new data of temporal stability of the DMS among Brazilian cisgender gay and bisexual men.

Strengths of the present study include: (a) the focus on understudied and under-recognized populations in the MD literature; (b) recruitment of a large sample of gay and bisexual men; and (c) use of best practices in translation and validation of body-image instruments [58]. Furthermore, to the best of our knowledge it is the first study to assess the temporal stability of the MDDI and DMS in a sample of gay and bisexual adult men. However, certain limitations also should be noted. First, due to the non-probabilistic sample, the results may not be generalizable to all Brazilian cisgender gay and bisexual men. Second, assessments were limited to self-report, which may reflect social bias. Finally, sample recruitment was carried out through social networks (i.e., Facebook^®^, Twitter^®^, and LinkedIn^®^), which may result in an overrepresentation of the sample, possibly limiting generalization.

## 5. Conclusions

Taken together, results of the present study provide support for MDDI and DMS as appropriate measures to assess muscularity concerns in Brazilian cisgender gay and bisexual adult men. These findings provide the foundation for expanding the drive for muscularity and muscle dysmorphia literature to more diverse populations. Future studies will be needed to yield further psychometric validation of the MDDI and the DMS among gender minorities (i.e., transgender and non-binary individuals, among others), and people from diverse racial or ethnic backgrounds (i.e., black, other ethnic origins) in Latin America.

## Figures and Tables

**Table 1:** Descriptive statistics and test of differences between the exploratory factor analysis (EFA) and confirmatory factor analysis (CFA) samples on demographics.

Variables	EFA Sample (*n* = 704)	CFA Sample (*n* = 705)	Test Result ^c^	Degrees of Freedom	*p*-Value
Age ^a^ (years)	27.23 (5.41)	26.68 (5.17)	1.942	1.407	0.052
BMI ^a^ (kg/m^2^)	26.11 (5.25)	25.87 (5.26)	0.845	1.407	0.398
Race/ethnicity ^b^	-	-	1.261	4	0.868
White	408 (57.95%)	400 (56.74%)	-	-	-
Brown	187 (26.56%)	201 (28.51%)	-	-	-
Black	95 (13.49%)	94 (13.33%)	-	-	-
Yellow	10 (1.43%)	7 (0.99%)	-	-	-
Indigenous	4 (0.57%)	3 (0.43%)	-	-	-
Sexual Orientation ^b^	-	-	0.056	1	0.813
Gay	598 (84.94%)	602 (85.39%)	-	-	-
Bisexual	106 (15.06%)	103 (14.61%)	-	-	-

Note. EFA = exploratory factor analysis; CFA = confirmatory factor analysis; BMI = body mass index. The categories suggested by the Brazilian Institute of Geography and Statistics [45] were used to classify race/ethnicity. Namely, these were: White (*branco*), Brown (*pardo* or “Mixed”), Black (*preto*), Yellow (*amarelo*), Indigenous (*indígena*), and “Other” (*outro*). ^a^ Results expressed by mean and standard deviation—*M* (*SD*). ^b^ Results expressed in absolute and relative frequency. ^c^ Test result for numerical data (Student’s *t*-test) or categorical data (chi-squared test).

**Table 2:** Descriptive statistics and factor loadings for EFA of the Drive for Muscularity Scale (DMS) of Brazilian cisgender gay and bisexual adult men.

DMS Items/Brazilian Translation	Mean (*SD*)	Range	Subscales	
			MBI (*λ*)	MB (*λ*)
1. I wish I were more muscular./*Quero ser mais musculoso(a).*	4.56 (1.50)	1–6	**0.74**	0.15
7. I think I would feel more confident if I had more muscle mass./*Acho que me sentiria mais confiante se meus músculos fossem maiores.*	4.11 (1.70)	1–6	**0.79**	0.10
11. I think I would feel stronger if I gained a little more muscle mass./*Acho que me sentiria mais forte se eu ganhasse um pouco mais de massa muscular.*	4.24 (1.71)	1–6	**0.74**	0.15
13. I think that my arms are not muscular enough./*Acho que meus braços não são musculosos o bastante.*	4.17 (1.71)	1–6	**0.93**	−0.10
14. I think that my chest is not muscular enough./*Acho que meu tórax não é musculoso o bastante.*	4.25 (1.71)	1–6	**0.88**	−0.13
15. I think that my legs are not muscular enough./*Acho que minhas pernas não são musculosas o bastante.*	3.60 (1.86)	1–6	**0.74**	−0.10
2. I lift weights to build more muscle./*“Levanto peso” para desenvolver meus músculos.*	3.39 (1.95)	1–6	0.05	**0.75**
3. I use protein or energy supplements./*Uso suplementos proteicos ou energéticos.*	2.43 (1.83)	1–6	−0.12	**0.91**
4. I drink weight gain or protein shakes./*Tomo shakes de proteína ou de ganho de massa.*	2.22 (1.75)	1–6	−0.13	**0.90**
5. I try to consume as many calories as I can in a day./*Diariamente, tento consumir o máximo de calorias possíveis.*	1.53 (1.53)	1–6	0.05	**0.51**
6. I feel guilty if I miss a weight-training session./*Sinto culpa se perco um treino de musculação.*	2.29 (1.60)	1–6	0.07	**0.73**
8. Other people think I work out with weights too often./*Os outros acham que eu faço exercícios com peso com muita frequência.*	1.96 (1.48)	1–6	-0.09	**0.73**
10. I think about taking anabolic steroids./*Penso em tomar esteroides anabolizantes.*	1.86 (1.46)	1–6	0.14	**0.43**
Eigenvalue			5.47	2.19
Explained variance (subscales)			30.2%	28.7%
Total explained variance			58.9%	

Note. *n* = 704; EFA = exploratory factor analysis; DMS = Drive for Muscularity Scale; *SD* = standard deviation; MBI = Muscularity-Oriented Body Image; MB = Muscularity-Oriented Behaviors; *λ* = factor loading. Values in **bold** indicate that an item loaded onto the corresponding factor.

**Table 3:** Descriptive statistics and factor loadings for EFA of the Muscle Dysmorphic Disorder Inventory (MDDI) for Brazilian cisgender gay and bisexual adult men.

MDDI Items/Brazilian Translation	Mean (*SD*)	Range	Subscales		
			DFS (*λ*)	AI (*λ*)	FI (*λ*)
1. I think my body is too small./*Eu acho que meu corpo é muito pequeno.*	2.06 (1.22)	1–5	**0.63**	0.02	0.07
4. I wish I could get bigger./*Eu gostaria de poder ficar maior.*	2.78 (1.40)	1–5	**0.77**	0.12	0.07
5. I think my chest is too small./*Eu acho que meu peitoral é muito pequeno.*	2.66 (1.45)	1–5	**0.76**	0.03	0.09
6. I think my legs are too thin./*Eu acho que minhas pernas são muito finas.*	2.29 (1.46)	1–5	**0.71**	0.10	0.01
8. I wish my arms were bigger./*Eu gostaria que meus braços fossem maiores.*	3.39 (1.38)	1–5	**0.65**	0.14	0.11
2. I wear loose clothing so that people cannot see my body./*Eu uso roupas largas para que as pessoas não vejam meu corpo.*	2.28 (1.29)	1–5	0.06	**0.70**	0.07
3. I hate my body./*Eu odeio meu corpo.*	2.42 (1.28)	1–5	0.15	**0.81**	0.02
7. I feel like I have too much body fat./*Eu sinto que eu tenho muita gordura corporal.*	3.09 (1.46)	1–5	0.29	**0.63**	0.15
9. I am very shy about letting people see me with my shirt off./*Eu tenho muita vergonha de deixar as pessoas me verem sem camisa.*	3.15 (1.42)	1–5	0.13	**0.78**	0.04
10. I feel anxious when I miss one or more workout days./*Eu me sinto ansioso(a) quando perco um ou mais dias de treino.*	2.40 (1.44)	1–5	0.01	0.07	**0.77**
11. I pass up social activities (e.g., watching football games, eating dinner, going to see a movie, etc.) with friends because of my workout schedule./*Eu deixo de fazer atividades sociais (por exemplo: assistir a um jogo de futebol, jantar, ir assistir a um filme, etc.) com amigos por causa da minha rotina de treinos.*	1.58 (.99)	1–5	0.01	0.08	**0.76**
12. I feel depressed when I miss one or more workout days./*Eu me sinto deprimido(a) quando perco um ou mais dias de treino.*	1.91 (1.24)	1–5	0.01	0.01	**0.89**
13. I pass up chances to meet new people because of my workout schedule./*Eu deixo de conhecer pessoas novas por causa da minha rotina de treinos.*	1.36 (.82)	1–5	0.02	0.02	**0.66**
Eigenvalue		3.32	1.46	2.48	3.32
Explained variance (subscales)		20.2%	16.7%	19.0%	20.2%
Total explained variance			55.9%		

Note. *n* = 704; EFA = exploratory factor analysis; MDDI = Muscle Dysmorphic Disorder Inventory; *SD* = standard deviation; DFS = Drive for Size; AI = Appearance Intolerance; FI = Functional Impairment; *λ* = factor loading. Values in **bold** indicate that an item loaded onto the corresponding factor.

**Table 4:** Descriptive statistics and bivariate correlations between the MDDI and DMS, and convergent measures.

Variables	*M* (*SD*)	Range	1.	2.	3.	4.	5.	6.	7.	8.	9.	10.	11.	12.
1. DMS MBI	17.23 (9.13)	7–42	-											
2. DMS MB	24.85 (8.72)	6–36	0.45 ***	-										
3. MDDI DFS	13.61 (5.63)	5–25	0.71 ***	0.39 ***	-									
4. MDDI AI	10.66 (4.25)	4–20	0.25 ***	−0.07	−0.05	-								
5. MDDI FI	7.50 (4.04)	4–20	0.38 ***	0.68 ***	.25 ***	0.18 ***	-							
6. EDE-Q	2.22 (1.30)	0–6	0.27 ***	0.12 **	−0.06	0.75 ***	0.32 ***	-						
7. SATAQ-4R BF	2.69 (1.33)	1–5	−0.03	−0.08 *	−0.25 ***	0.45 ***	0.07	0.56 ***	-					
8. SATAQ-4R MUS	3.48 (1.17)	1–5	0.76 ***	0.53 ***	0.57 ***	0.14 ***	0.43 ***	0.25 ***	0.03	-				
9. SATAQ-4R GA	4.35 (0.79)	1–5	0.34 ***	0.19 ***	0.18 ***	0.17 ***	0.22 ***	0.28 ***	0.12 **	0.34 ***	-			
10. SOBBS OP	3.50 (1.00)	1–5	0.53 ***	0.19 ***	0.31 ***	0.55 ***	0.30 ***	0.55 ***	0.27 ***	0.46 ***	0.38 ***	-		
11. SOBBS VB	2.32 (0.79)	1–5	0.38 ***	0.17 ***	0.21 ***	0.44***	0.250 ***	0.46 ***	0.26 ***	0.42 ***	0.28 ***	0.64 ***	-	
12. BAS-2	3.29 (0.88)	1–5	−0.33 ***	0.07	−0.11 **	−0.77 ***	0.12 **	−0.62 ***	−0.35 ***	−0.22 ***	−0.18 ***	−0.54 ***	−0.52 ***	-

Note. *n* = 705. M = mean; SD = standard deviation; DMS = Drive for Muscularity Scale; MB = Muscularity-Oriented Behaviors subscale; MBI = Muscularity-Oriented Body Image subscale; MDDI = Muscle Dysmorphic Disorder Inventory; DFS = Drive for Size subscale; AI = Appearance Intolerance subscale; FI = Functional Impairment subscale; EDE-Q = Eating Disorder Examination—Questionnaire; SATAQ-4R = Sociocultural Attitudes Towards Appearance Questionnaire 4 Revised; BF = Internalization Thin/Low Body Fat subscale; MUS = Internalization Muscularity subscale; GA = Internalization General Attractiveness subscale; SOBBS = Self-Objectification Beliefs and Behaviors Scale; SOBBS OP = Observer’s Perspective subscale; SOBBS VB = Valuing the body above other attributes and qualities and the body capable of representing itself subscale; BAS-2 = Body Appreciation Scale 2. * *p* < 0.05, ** *p* < 0.01, *** *p* < 0.001.

**Table 5:** Internal consistency and test–retest reliability of the DMS and the MDDI for Brazilian cisgender gay and bisexual adult men.

Variables	McDonalds’ *ω* (Test) CI (95%]	McDonalds’ *ω* (Retest) CI (95%]	Spearman Correlation (*Rho*)	Intraclass Correlation CI (95%]
DMS MB	0.87 [0.85, 0.90]	0.88 [0.86, 0.91]	0.93 ***	0.93 [0.91, 0.94] ***
DMS MBI	0.93 [0.91, 0.94]	0.94 [0.93, 0.95]	0.86 ***	0.86 [0.83, 0.89] ***
MDDI DFS	0.85 [0.82, 0.88]	0.84 [0.80, 0.88]	0.87 ***	0.87 [0.84, 0.90] ***
MDDI AI	0.78 [0.73, 0.83]	0.79 [0.75, 0.84]	0.89 ***	0.89 [0.86, 0.91] ***
MDDI FI	0.89 [0.87, 0.92]	0.86 [0.82, 0.90]	0.80 ***	0.84 [0.80, 0.87] ***

Note. *N*_test_ = 705. *N*_retest_ = 188. CI = confidence interval; DMS = Drive for Muscularity Scale; MB = Muscularity-Oriented Behaviors subscale; MBI = Muscularity-Oriented Body Image subscale; MDDI = Muscle Dysmorphic Disorder Inventory; DFS = Drive for Size subscale; AI = Appearance Intolerance subscale; FI = Functional Impairment subscale. *** *p* < 0.001.

## Data Availability

The data presented in this study are available on request from the corresponding author. The data are not publicly available due to not obtaining consent from respondents to publish the data.

## Supplementary Materials

The following supporting information can be downloaded at: https://www.mdpi.com/article/10.3390/ijerph20020989/s1. Figure S1: Confirmatory factor analysis results (standardized factor loading and residuals) of the Muscle Dysmorphic Disorder Inventory (MDDI) for Brazilian cisgender gay and bisexual adult men. Figure S2: Confirmatory factor analysis results (standardized factor loading and residuals) of the Drive for Muscularity Scale (DMS) for Brazilian cisgender gay and bisexual adult men.
