# Supplementary material for: Psychometric Evaluation of the Drive for Muscularity Scale and the Muscle Dysmorphic Disorder Inventory among Brazilian Cisgender Gay and Bisexual Adult Men
Source: Int J Environ Res Public Health. 2023 Jan 5;20(2):989. doi: 10.3390/ijerph20020989 (PMC9858959; doi:10.3390/ijerph20020989)

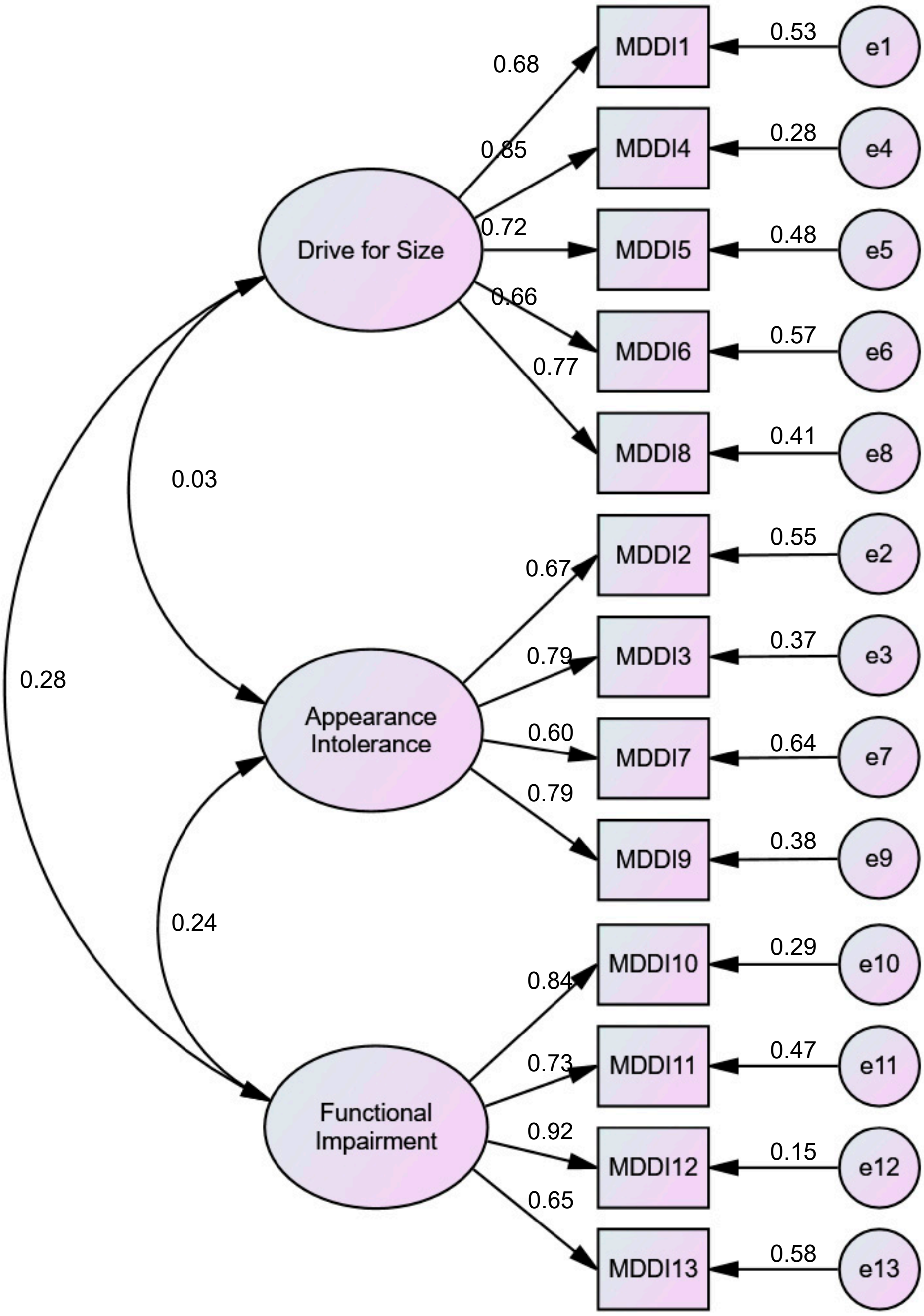

Figure S1. Confirmatory factor analysis results (standardized factor loading and residuals) of the Muscle Dysmorphic Disorder Inventory (MDDI) for Brazilian cisgender gay and bisexual adult men.

Figure S2. Confirmatory factor analysis results (standardized factor loading and residuals) of the Drive for Muscularity Scale (DMS) for Brazilian cisgender gay and bisexual adult men.

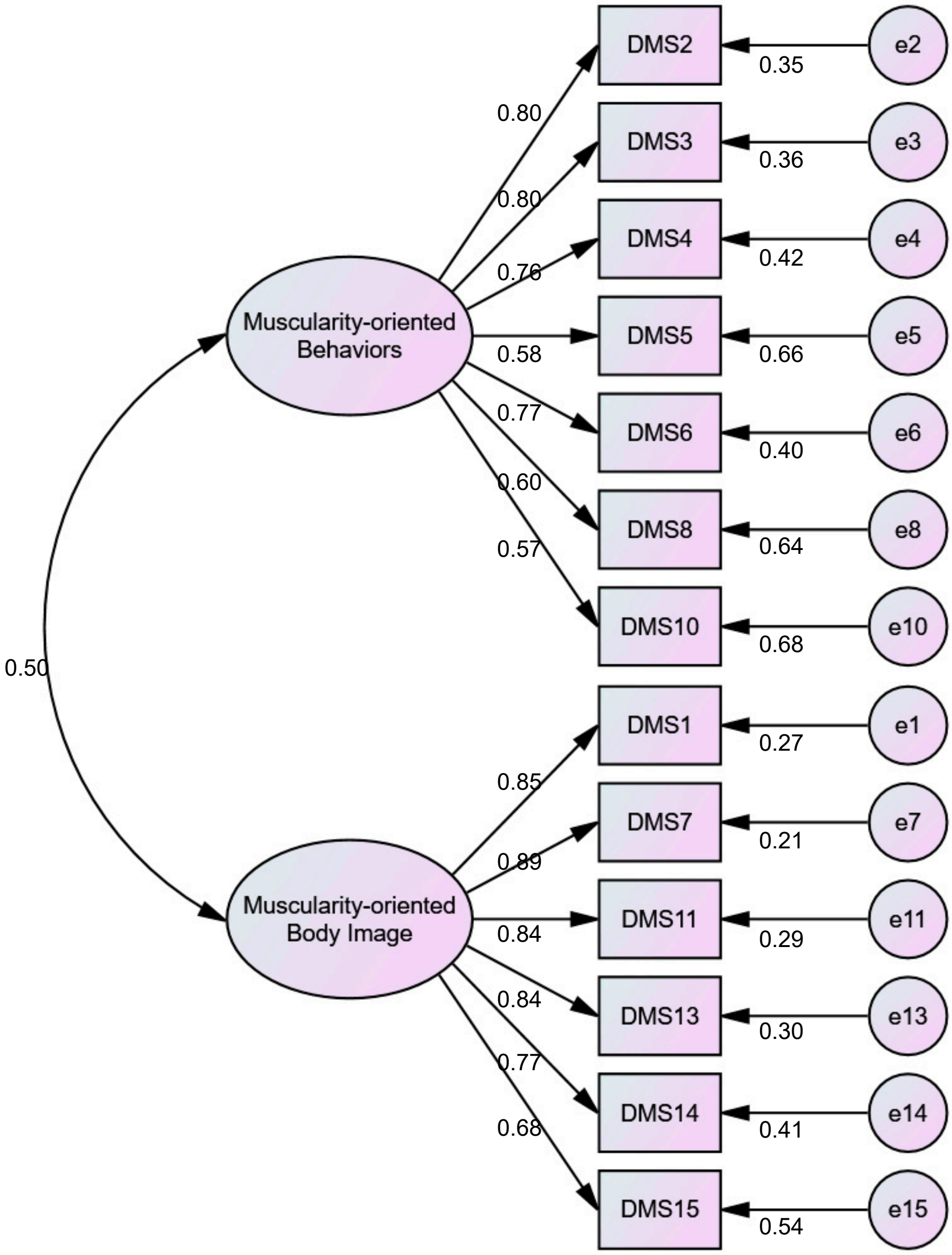

Supplement: Supplementary file 1 [file ijerph-20-00989-s001.zip › ijerph-2061317-supplementary.pdf]
